# Remodeling of tRNA modification in *Trypanosoma cruzi* life forms

**DOI:** 10.1371/journal.ppat.1014249

**Published:** 2026-05-26

**Authors:** Herbert Guimaraes de Sousa Silva, Janaina de Freitas Nascimento, Marilene Souza Braga, Ana Paula de Jesus Menezes, Ariel M. Silber, Matthew K. Waldor, Julia Pinheiro Chagas da Cunha, Satoshi Kimura

**Affiliations:** 1 Laboratório Ciclo Celular, Instituto Butantan, São Paulo, Brazil; 2 Departamento de Microbiologia, Imunologia e Parasitologia, Escola Paulista de Medicina da Universidade Federal de São Paulo, São Paulo, Brazil; 3 Department of Microbiology and Immunology, Cornell University College of Veterinary Medicine, Ithaca, United States of America; 4 Laboratório de Bioquímica de Tryps – LaBTryps, Departamento de Parasitologia, Instituto de Ciências Biomédicas - Universidade de São Paulo, São Paulo, São Paulo, Brazil; 5 Division of Infectious Diseases, Brigham and Women’s Hospital, Boston, United States of America; 6 Department of Microbiology, Harvard Medical School, Boston, United States of America; 7 Howard Hughes Medical Institute, Boston, United States of America; California State University Fullerton, UNITED STATES OF AMERICA

## Abstract

*Trypanosoma cruzi,* the etiological agent of Chagas disease, infects millions of people in the Americas. This parasite undergoes drastic changes in its morphology and metabolism between infective and noninfective forms through global remodeling of its proteome. Chemical modification of tRNA (tRNA modification) contributes to the control of protein expression by modulating the codon decoding process. However, knowledge of tRNA modification profiles, the enzymes that create modifications and their regulation in different cellular conditions is largely restricted to relatively few model organisms. Here, we profile tRNA modifications in both infective and noninfective forms of *T. cruzi* to probe their dynamic changes. Genome mining of tRNA modifying enzymes identified 65 putative tRNA-modifying enzymes in *T. cruzi* for 27 species of tRNA modifications, most of which were detected in *T. cruzi* tRNA by liquid chromatography mass spectrometry analyses. tRNA sequencing detected reverse transcription-derived signatures at 170 sites in *T. cruzi* tRNAs that are likely derived from 19 tRNA modifications. tRNA modifications and tRNA modification enzymes are differentially modulated across the life stages of *T. cruzi.* We found that hydroxywybutosine (OHyW) at position 37 on tRNA^Phe(GAA)^ had a reduced level in the infective form (metacyclic trypomastigote) and the associated modification enzyme Tyw1a exhibited reduced expression in this stage. Knockout of Tyw1a increased the differentiation from epimastigote (noninfective form) to metacyclic trypomastigote, suggesting that changes in OHyW37 modification levels alter the rate of metacyclogenesis. Overall, our findings suggest that tRNA modification changes during the life stages of *T. cruzi* contribute to the differentiation of this parasite.

## Author summary

*Trypanosoma cruzi* infects millions of people worldwide. This protozoan exhibits polycistronic transcription for coding sequences and undergoes a complex life cycle involving noninfective and infective forms. A key question in the *T. cruzi* biology is how this parasite predominantly regulates gene expression at the post-transcriptional level during its differentiation. tRNA modifications has become increasingly recognized as an alternative layer of post-transcriptional regulation for gene expression in many organisms by modulating the decoding ability and tRNA stability. Here, we present the first profiling of global tRNA modifications in *T. cruzi*, revealing dynamic changes associated with its distinct life forms. These include a reduction in Tyw1a expression as well as in its associated tRNA modification at position 37 of tRNA^Phe(GAA)^, hydroxywybutosine (OHyW37), upon differentiation from the non-infective (EPI) to the infective (MT) form, which is facilitated by the disruption of Tyw1a. Our findings highlight that tRNA modification changes might play an important role in facilitating parasite differentiation. Additionally, the tRNA epitranscriptomic modulation identified in this study across *T. cruzi* life forms offers promising avenues to explore the role of tRNA epitranscriptome in parasite differentiation.

## Introduction

The Trypanosomatidae is a family of protozoa that includes human pathogens such as *Trypanosoma brucei*, *Leishmania major*, and *Trypanosoma cruzi*. *T. cruzi*, the causative agent of Chagas disease, poses a significant global health concern, impacting approximately seven million people worldwide [1]. The process of differentiation between *T. cruzi* life forms involves alterations in their morphologies, metabolic patterns, and gene and protein expression [2–7].

*T. cruzi* alters global gene expression profiles to survive and adapt under different conditions, such as variations in pH, osmolarity, temperature, and high levels of oxidative stress [8–10]. Since this parasite relies on polycistronic transcription where control of expression of individual genes is limited, the regulation of protein expression is predominantly governed by post-transcriptional mechanisms [11]. Recently, we suggested that this parasite may harbor a new layer of regulatory control of protein expression based on tRNA availability and anticodon::codon pairing modes. In this system, highly expressed mRNAs show greater coadaptation to tRNA abundance and preferentially use codons that favor Watson-Crick pairing. In contrast, less expressed mRNAs exhibit lower coadaptation to tRNA abundance and tend to use codons that rely more on wobble pairing with tRNAs [12].

tRNA modifications have become increasingly recognized as an important layer of regulation for protein synthesis. Through modulating the decoding ability and stability of tRNAs, tRNA modifications play a key role in the survival of organisms [13–17]. Several studies have linked the dynamic changes in tRNA modification to critical biological processes, such as cancer progression, bacterial virulence, and the ability of organisms to adapt in extreme or hostile environments [18–21]. The impact of tRNA modification on the efficiency of translating specific classes of proteins depends on the type and position of specific modifications. Therefore, profiling types and positions of tRNA modifications is essential to understand their roles in translation modulation. tRNA modifications are present across all kingdoms of life but vary among organisms [22]. However, the profiles and functions of tRNA modifications are still largely undefined in many organisms.

Among Trypanosomatids, some tRNA modifications have been well studied in *T. brucei*, where they are reported to regulate the subcellular localization of tRNAs, codon recoding, and mitochondrial translation [23–29]. However, in both *T. brucei* and *T. cruzi*, the full repertoire of tRNA modifications and their global changes across different life stages have not been evaluated.

tRNA modification sites can be predicted and identified by multiple methods [30]. *In silico* search for homologs of known tRNA-modifying enzymes allows prediction of modifications based on known activities of tRNA-modifying enzymes in other organisms [31]. However, this method cannot predict the modifications that are not observed in other organisms or sites of modifications that can vary among different organisms due to the variations in the substrate specificity of the homologs of tRNA modifying enzymes. tRNA sequencing (tRNA-seq) enables unbiased rapid prediction of tRNA modification sites in all tRNA species, based on the detection of reverse transcription (RT)-derived signatures [32]. Some modifications on tRNAs inhibit Watson-Crick base pairing, which increases the frequency of misincorporation (MI) of incorrect bases and premature termination (TE) during the cDNA synthesis process [32]. The change of tRNA modification at the specific site can be detected by the changes in these RT-derived signatures.

Here, coupling mass spectrometry analysis, tRNA-seq and bioinformatic searches for tRNA-modifying enzyme, we profiled tRNA modifications in *T. cruzi*. tRNA modifications conserved in other eukaryotes as well as Trypanosoma specific signals were uncovered by tRNA-seq. Comparison of RT-derived signatures in tRNA-seq data from infective (metacyclic trypomastigote – MT, and tissue cultured trypomastigote - TCT) and noninfective (epimastigote-EPI) forms revealed that tRNA modification profiles are dynamic across *T. cruzi* differentiation. A subset of the dynamic changes of tRNA modification frequencies were correlated with changes in the expression levels of tRNA modifying enzymes. Finally, we found that the deletion of the tRNA modifying enzyme Tyw1a, which is associated with the OHyW37 modification pathway on tRNA^Phe^, increases parasite differentiation.

## Results

### *In silico* identification of tRNA modifying enzymes in Trypanosoma and predicting their essentiality and localization

**Fig 1:**
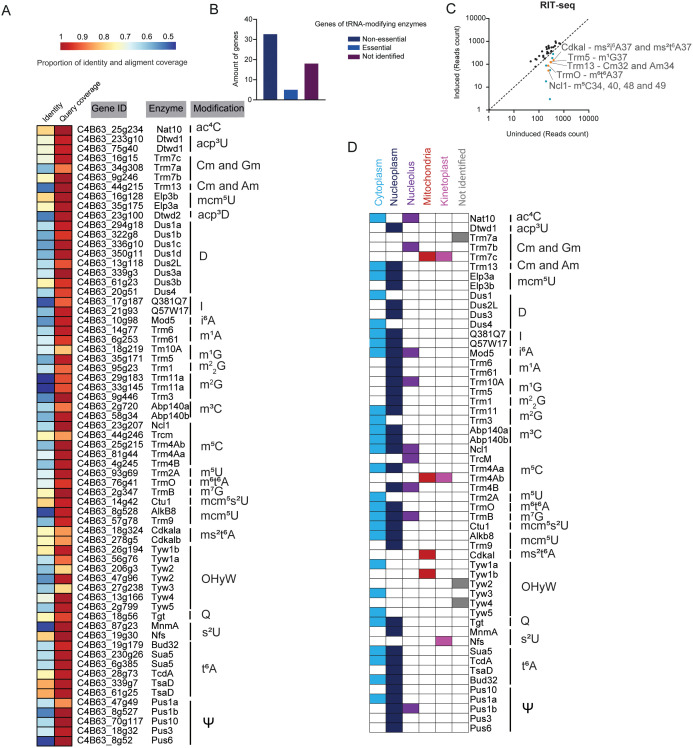
Identification, essentiality, and localization of tRNA-modifying enzymes in Trypanosoma. **A)** BLAST search of tRNA modifying enzymes in *T. cruzi*. Proportion of query coverage and identity between subject protein sequences from the *T. cruzi* genome (Dm28c – version 2018) and known tRNA-modifying enzymes listed in S1B Table. The heatmap displays the gene ID and enzyme names of the candidate homologs in *T. cruzi* identified. E-values ≤ 1e-10 were used as a threshold for candidate selection. **B**) Essentiality of tRNA-modifying enzymes in Trypanosoma. The number of tRNA-modifying enzyme genes identified in public RNA Interference Target Sequencing (RIT-seq) data [35] associated with *T. brucei* survival during the differentiation of procyclic to bloodstream trypomastigote forms. **C)** RIT-seq data identifying a subset of tRNA-modifying enzymes required for *T. brucei* fitness. Reads Per Five Million (RPFM) for loci of double-stranded RNA (dsRNA), which target tRNA-modifying enzymes via RNA interference (RNAi), are plotted under induced (y-axis) or non-induced (x-axis) conditions. A decrease in RPFM under induced conditions suggests that the target gene contributes to optimal growth of the parasites. tRNA-modifying enzymes showed lower RPFM upon induction (lower than the dashed line) are colored orange (statistically significant) and green (not statistically significant). Orange dots represent tRNA-modifying enzymes associated with loss of parasite fitness, while black and green dots show no significant effect. **D)** Subcellular localization of tRNA-modifying enzymes in the *T. brucei*: cytoplasm (light blue), nucleoplasm (dark blue), nucleolus (purple), mitochondria (red), and kinetoplast (pink). The cellular localization of some proteins, for which no public data is available (grey), is shown as ‘not identified’.

We compiled a list of 217 protein sequences of known and putative tRNA-modifying enzymes present in various eukaryotic and prokaryotic species, ranging from human to *Escherichia coli* (S1A Table). BLAST was employed to search for homologs of these tRNA modifying enzymes in the *T. cruzi* genome. Using an E-value of 1e-10 as a cutoff, we identified sixty-five homologs of tRNA-modifying enzymes in *T. cruzi*; these enzymes likely mediate 27 types of tRNA modifications, including dihydrouridinylation, thiolation, aminocarboxypropylation, methylation and acetylation (Fig 1A and S1B Table). Some tRNA modifying enzymes have multiple paralogs in *T. cruzi*. For example, the methyltransferase Abp140 from yeast (Gene ID = Q08641) has two orthologs in *T. cruzi*, Abp140a (C4B63_2g720) and Abp140b (C4B63_58g34) (Fig 1A and S1B Table). We added suffixes (a, b, or c) to the end of the enzyme name to distinguish the candidate paralogs identified in this study. All these tRNA modifying enzymes have homologs in *T. brucei* (S1C Table), which displays 94% similarity and 57% identity with the *T. cruzi* genome [33,34], suggesting that tRNA modification profiles are conserved in different species of Trypanosoma.

To gain further insights into the impact of tRNA-modifying enzymes on *Trypanosoma* fitness, we analyzed high-throughput phenotyping data, RNA Interference Target Sequencing (RIT-seq) data, available for *T. brucei* [35]. Among the fifty-six tRNA-modifying enzymes identified in *T. brucei*, no data is available for nineteen (34%) (Fig 1B and S1D Table). Of the enzymes with RIT-seq data, thirty-two (86%) were classified as non-essential, while five (14%) were considered essential during the *T. brucei* life stages, such as Trm13 and TrmO, involved with m^1^G37 and m^6^t^6^A37modifications, respectively (Fig 1C). These results indicate that specific tRNA modifications play a key role in *Trypanosoma* fitness.

tRNA modifying enzymes are known to be found at various subcellular locations. To evaluate their cellular localization, we interrogated the *T. brucei* genome-wide database of subcellular protein localization [36]. We found that most of the tRNA-modifying enzymes are in the nucleoplasm and in the cytoplasm (Fig 1D), whereas some enzymes are localized in organelles. Trm7c and Trm4Ab associated with Gm and m^5^C, respectively, are predicted to localize in both mitochondria and kinetoplast (Fig 1D). Curiously, we identified some paralogs, such as Trm4Aa and Trm4Ab, which exhibit distinct subcellular localization patterns, where Trm4Aa can be found in cytoplasm/nucleoplasm and Trm4Ab in mitochondria/kinetoplast. These observations suggest that these homologous enzymes participate in tRNA modification pathways in different cellular compartments, although their precise catalytic roles and targets remain to be experimentally validated.

### Profiling the repertoire and sites of tRNA modifications in *T. cruzi*

**Fig 2:**
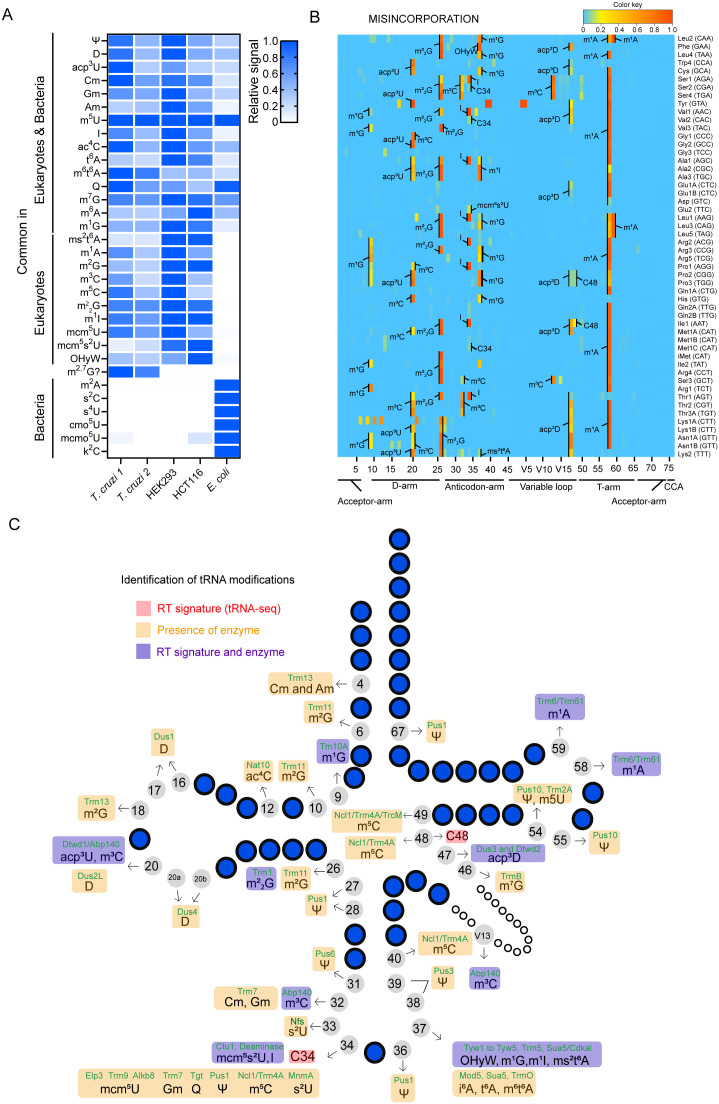
The tRNA modification profiles in *T. cruzi* EPI life form. **A)** LC-MS analysis of total tRNA fraction from *T. cruzi*, human cell lines (HEK293 and HCT116), and *E. coli*. Individual signal intensities were normalized to the m^5^U signal, and then relative values to the maximum value across samples for each modified nucleoside are shown. Two biological replicates for *T. cruzi* samples and one replicate for HEK293, HCT116, and *E. coli* samples are shown. Chromatograms and source data are provided in S1 Fig and S3 Table. **B)** Heatmaps display misincorporation frequencies calculated from tRNA-seq results from the *T. cruzi* EPI form. Predicted tRNA modifications on RT-derived signatures are shown. The position and species of tRNA are indicated in the x- and y-axis, respectively. **C)** Overview of all tRNA modifications detected by tRNA-seq and predicted based on the presence of tRNA-modifying enzymes. The tRNA-modifying enzymes are highlighted in green above their respective modifications. Modifications predicted by both RT-derived signature and the presence of the corresponding enzyme are shown in purple.

To profile the repertoire of tRNA modifications, we analyzed the total nucleosides of *T. cruzi* tRNAs by liquid chromatography mass spectrometry (LC-MS). For the references, tRNAs from human cell lines (HEK293T and HCT116) and *Escherichia coli* were also analyzed. tRNA fractions were enzymatically digested into nucleosides and subject to LC-MS. We successfully detected 32 modifications in either *T. cruzi*, human cell lines, or *E. coli* (Fig 2A, S1, S3 Table). Among them, 26 modified nucleosides were detected in *T. cruzi*, covering most modification types predicted by *in silico* search for tRNA-modifying enzymes. Despite the phylogenetic distance, *T. cruzi* shares most tRNA modifications with humans but not with bacteria. One notable *T. cruzi*-specific signal was the dimethyl-guanosine peak (Figs 2A, S1 Fig) in addition to m^2^_2_G peak observed in human cell lines. This peak may correspond to the other reported dimethylguanosine, m^2,7^G, or a totally novel modification.

To profile tRNA modification sites in *T. cruzi*, we employed tRNA-seq. tRNA from exponentially growing noninfective state (EPI form) was purified and sequenced and exhibited high sequence quality and read mapping across replicates (S2A-S2B Fig). Several tRNA modifications perturb reverse transcription during cDNA synthesis, which leads to the misincorporation of incorrect bases and premature termination of reverse transcription, which we call reverse transcription-derived signatures (RT-derived signatures). Therefore, RT-derived signatures detected in tRNA-seq data likely correspond to modified sites as previously published in prokaryotes [32,37]. The misincorporation and termination signals in *T. cruzi* tRNAs are shown in Figs 2B and S3A and S3B.

Many RT-derived signatures are consistent with conserved modification sites in eukaryotes, such as human and yeast [38]. The sites and modifications predicted in the *T. cruzi* profiles include m^1^G at position 9, m^3^C and acp^3^U at position 20, m^2^_2_G at position 26, m^3^C at position 32, 20 and V13, I at position 34, m^1^G, OHyW, and ms^2^t^6^A at position 37, and m^1^A at position 58 and 59 (Fig 2B). Furthermore, the homologs of tRNA modifying enzymes that synthesize these modifications were also identified in *T. cruzi* (Figs 1A, 2C and S1B Table). These observations support the assignment of tRNA modifications to *T. cruzi* RT-derived signatures.

We detected misincorporation and termination signatures that are derived from the modifications observed in *T. brucei*, at position 47 in twenty-two tRNA species, such as tRNA^Lys1A(CTT)^, tRNA^Asn1B (GTT)^, tRNA^Lys2 (TTT)^ (Fig 2B and 2C). In *T. brucei*, tRNA^Lys(TTT)^ is modified at position 47 by a unique modification acp^3^D [29], suggesting that the RT-derived signature detected in *T. cruzi* is likely derived from acp^3^D.

Some RT-derived signatures in *T. cruzi* are likely derived from the modifications in mitochondrial tRNAs. Unlike mammals and yeast, Trypanosoma utilizes nuclear-encoded tRNAs not only in the cytoplasm but also in mitochondria [39,40]. A fraction of tRNAs is imported into mitochondria and some of them are further modified. Thus, some of the RT-derived signatures are likely derived from the fraction of mitochondrial tRNAs. In *T. brucei*, tRNA^Trp4(CCA)^ is imported into mitochondria, and its wobble position is edited from C-to-U leading to decoding the termination codon (UGA) as tryptophan [41]. The misincorporation signals at position 34 observed in *T. cruzi* tRNA^Trp4(CCA)^ (Fig 2B) suggest that *T. cruzi* tRNA^Trp4(CCA)^ is also edited at position 34 for UGA codon recoding. Additionally, the wobble position of tRNA^Met^ is modified into formyl cytidine (f^5^C) in mitochondria in mammals to decode two methionine codons, AUG and AUA, in mitochondria [42]. Misincorporation signals at position 34 in tRNA^Met1B(CAT)^, tRNA^Met1C(CAT)^ (Fig 2B), and the presence of a homolog of ALKBH1 (C4B63_7g420), which synthesizes f^5^C, in *T. cruzi* suggests that f^5^C is utilized to expand the codon decoding ability of tRNA^Met^ in Trypanosoma mitochondria.

In addition, we observed some RT-derived signatures in tRNAs from *T. cruzi* that are not observed in other organisms, such as misincorporation at position 34 in tRNA^Ser2(CGA)^ and tRNA^Val2(CAC)^, and at C48 in tRNA^Pro2(CGG)^, tRNA^Pro3(TGG)^, and tRNA^Ile1(AAT)^. These signatures likely represent modifications exclusively present in *T. cruzi* and other trypanosomatids, while orthogonal validation is necessary.

While we found *T. cruzi* homologs of tRNA-modifying enzymes *in silico* in *T. cruzi*, some expected tRNA modifications were undetectable by tRNA-seq due to the absence of RT-derived signatures. We found 42 enzymes that likely synthesize tRNA modifications in *T. cruzi,* but their modification sites were not detected by tRNA-seq, as they do not inhibit Watson-Crick base pairing, such as D20, m^5^U54, Q34 and mcm^5^U34 (Fig 2B, S1B and S2B Table).

### tRNA modification levels change in different *T. cruzi* life forms

tRNA modification levels can change under different environmental conditions for optimizing the synthesis of specific proteins [32,43]. *T. cruzi* changes their life forms in the face various environmental pressures; the different forms include a noninfective replicative EPI form, and infective and cell-cycle arrested TCT and MT forms [7–10,44]. We hypothesized that differential expression of tRNA modifying enzymes could contribute to the optimization of protein expression profiles in different *T. cruzi* life forms via alteration of tRNA modification profiles. To test this idea, we investigated whether tRNA-modifying enzymes are differently expressed in their replicative (EPI) and non-replicative (MT) forms using publicly available Ribo-seq data [7].

**Fig 3:**
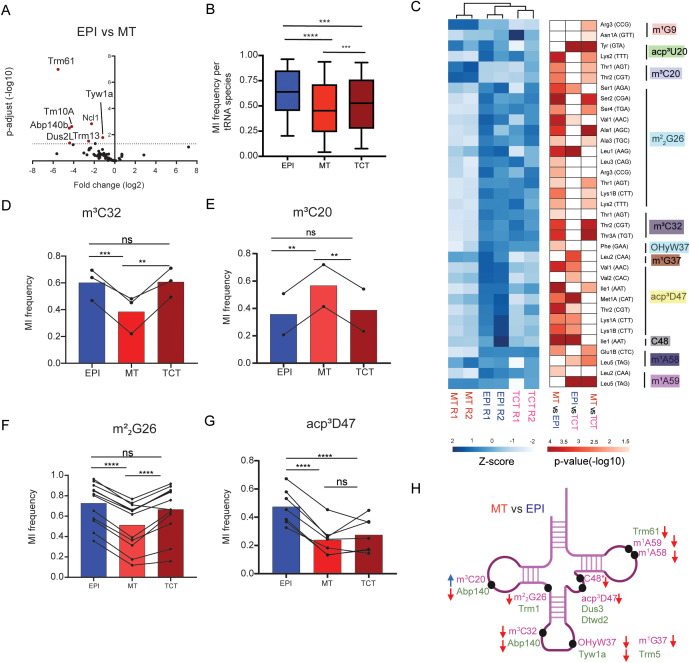
Changes in the expression levels of tRNA-modifying enzymes and the frequencies of tRNA modification across different stages of *T. cruzi.* **A)** Volcano plot showing the fold change (log_2_) and p-adjust (log_10_) of the expression levels of tRNA-modifying enzymes in EPI compared to MT using Ribo-seq data [7]. **B)** Global changes of misincorporation rates in tRNA-seq data derived from *T. cruzi* life forms (EPI, MT and TCT, n = 170). Statistical significance tests (p-value ≤ 0.05) were performed with the two-way ANOVA test corrected by Tukey’s multiple comparison tests (for adjusted p values: **** = 0.0001; *** = 0.001; ** = 0.01; * = 0.05; ns = not significant). **C)** Differential tRNA modification frequencies among EPI, MT, and TCT forms. The heatmaps illustrate the z-scores of misincorporation frequencies (left) and p-values from the indicated comparisons of the misincorporation frequencies derived from tRNA modifications (right). A two-way ANOVA test was employed to identify significantly differentially tRNA modifications containing a fold change of>= 20% (arbitrary threshold) between life forms. **D, E, F)** Misincorporation frequency of positions that show differential abundance in at least a set of two *T. cruzi* life forms. Data points represent the average of two replicates, and results of the same tRNA species are connected with lines. Bars represent the average values of multiple tRNA species. **D**) m^3^C32 (3 tRNA species), **E**) m^3^C20 (2 tRNAs species), **F)** m^2^_2_G26 (12 tRNA species), and **G)** acp^3^D47 (7 tRNA species). Statistical significance tests (adjusted p-value ≤ 0.05) were performed with the two-way ANOVA corrected by Tukey’s multiple comparisons test (for adjusted p values: **** = 0.0001; *** = 0.001; * = 0.05; ns = not significant). **H)** Illustration containing the abundance of tRNA modification and their corresponding modifying enzymes (Trm61, Dus3, Dtwd2, Trm5, Tyw1a, Abp140a, Abp140b, Trm1) in *T. cruzi*’s life forms. The tRNA modifying enzymes are shown in green, positioned below their respective catalyzed modifications. The red arrow indicates downregulation of tRNA modifying enzymes or tRNA modification in MT compared to EPI, while blue arrow indicates upregulation in MT. Enzymes without arrows have similar abundance in both EPI and MT.

Among the sixty-five *T. cruzi* tRNA-modifying enzymes, seven (10%) were differentially expressed between EPI and MT forms (Fig 3A and S1E Table). In the non-replicative MT form, all seven of these transcripts were downregulated by more than 2-fold (Fig 3A). Interestingly, we observed different trends in the expression of multiple paralogs of tRNA-modifying enzymes. The Abp140a and Abp140b are predicted to be localized at both cytoplasm and nucleoplasm, and only Abp140b are differently regulated in the EPI and MT forms (Fig 3A). Abp140a shows similar expression levels in EPI and MT cells, while Abp140b exhibits a drastic ~21-fold reduction in expression in MT cells, suggesting that the frequency of m^3^C synthesized by Abp140b specifically decreased in MT form.

Biosynthesis of wyosine (imG) and its derivatives, including wybutosine (yW) and hydroxywybutosine (OHyW) occurs in the cytoplasm of most eukaryotes. In contrast, in *T. brucei*, two independent biosynthesis pathways for wyosine derivatives are found in the cytoplasm (OHyW) and mitochondria (imG) [45]. Like *T. brucei*, *T. cruzi* encodes two paralogous Tyw1 loci, Tyw1a and Tyw1b, which are predicted to localize in the cytoplasm and mitochondria respectively (Fig 1D), suggesting that *T. cruzi* also possesses two parallel biosynthesis pathways for wyosine derivatives in these two cellular compartments. The level of cytoplasmic Tyw1a was decreased in EPI compared to in MT cells by 2.3-fold, while mitochondrial Tyw1b had similar expression levels in both life forms. Since these enzymes are presumably involved in the formation of wyosine derivatives, such as wybutosine and hydroxywybutosine at 37 in tRNA^Phe^, the differential expression of Tyw1a may contribute to stage-specific regulation of OHyW levels in cytoplasmic tRNA.

Next, to track the changes in the frequency of tRNA modification in different life forms of *T. cruzi*, we conducted tRNA-seq in EPI, MT and TCT forms (S2 Fig). Using misincorporation frequency as a readout of tRNA modification frequency, we tracked the changes of nineteen tRNA modifications at 170 positions across all tRNA species (S2C Table). The misincorporation frequencies showed high correlations among two replicates of individual parasite stages (S3C Fig). Overall, changes in the frequency of tRNA modifications were observed among MT, TCT, and EPI forms (Fig 3B). The misincorporation frequencies of tRNA modifications from EPI cells were more similar to TCT than MT cells (Fig 3C). When analyzing individual sites, we observed changes across different types of tRNA modifications. The frequency of 12 modifications, including m^2^_2_G26, m^3^C32 and OHyW37, was comparable between EPI and TCT but reduced in MT in many tRNA species (Fig 3C-3D and Fig 3F), whereas the frequency of some tRNA modifications was increased in MT (m^3^C20 in tRNA^Thr2(CGT)^ and tRNA^Thr1AGT^) compared to other forms (Fig 3C and 3E). Interestingly, a prominent increase in the frequencies of acp^3^D47 modification (with approximately 1.5- to 3-fold) was observed in several tRNAs, such as tRNA^Val1 (AAC)^, tRNA^Lys1A (CTT)^, tRNA^Thr2 (GGT)^, tRNA^Met1A (CAT)^ in the EPI compared to MT and TCT forms (Fig 3C and 3G). The acp^3^U47 is reported to promote thermal stability on tRNA [46], suggesting that the increased acp^3^D47 on tRNAs in proliferative forms could contribute to increased thermostability in proliferative forms.

The changes in tRNA modifications are likely at least partly caused by the differential expression of tRNA modifying enzymes in different life forms. Three tRNA-modifying enzymes downregulated in MT (Tyw1a, Abp140b, and Trm61) catalyze modifications (OHyW37, m^3^C32, m^1^A58, and m^1^A59) that exhibit a detectable reduction in their frequency in the MT form in tRNA-seq data (Fig 3C and 3H), suggesting that the tRNA modification frequency is controlled by the regulation of the tRNA modifying enzyme expression. However, some significant reduction in tRNA modification levels was not explained by the expression level of their corresponding tRNA modifying enzymes. For example, the level of m^3^C20 increased in MT compared to EPI, while the expression levels of its modifying enzymes (Abp140a or Abp140b) do not change accordingly in the life forms. These results might suggest that an alternative mechanism controls the m^3^C20 levels in MT, if steady-state protein measurements confirm a reduction similar to that observed in the Ribo-seq data.

### Knockout of Tyw1a promotes *T. cruzi* Differentiation

Next, we investigated whether the tRNA modifications and their associated tRNA-modifying enzymes that are differentially regulated across life stages influence *T. cruzi* differentiation. The tRNA-seq data showed that the misincorporation signal corresponding to the OHyW37 modification in MT decreased by approximately 1.23-fold compared to EPI, while the level of the Tyw1a enzyme in MT decreased by approximately 2-fold relative to EPI. Tyw1a, also known as Tyw1L, is essential for the formation of hydroxywybutosine (OHyW37) on tRNA^Phe^ and for growth in low-glucose medium in *T. brucei* [45]. *T. cruzi* bears the other homologs required for OHyW synthesis (Tyw2, Tyw3, Tyw4, and Tyw5). Furthermore, mass spec analysis confirmed that a signal corresponding to OHyW (*m/z* 525.1945 ⟶ 393.1523) was detected in the nucleoside pool of *T. cruzi* tRNA but not in that of *E. coli* (Figs 2A and S1), strongly supporting that *T. cruzi* also has OHyW in tRNA^Phe(GAA)^. In *T. cruzi*, proliferation arrest under nutrient-depleted conditions is crucial for metacyclogenesis, i.e., the differentiation from EPI to MT. Given that Tyw1a supports optimal growth of *T. brucei* under starvation condition, we hypothesized that Tyw1a suppresses differentiation from EPI to MT in *T. cruzi.*

**Fig 4:**
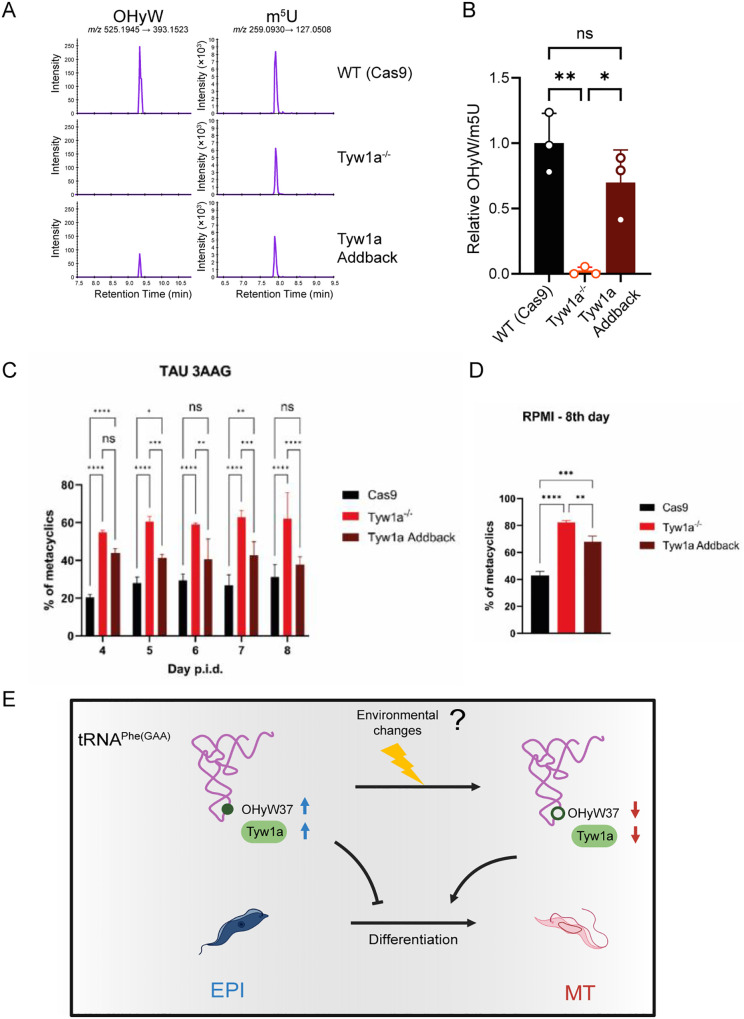
Tyw1a knockout facilitated *T. cruzi* differentiation. **A, B)** Validating the causal link between OHyW and Tyw1a. The total tRNA fraction was digested into nucleosides and analyzed by LC-MS with tSIM mode. **A**) Mass chromatograms detecting OHyW and m^5^U in three parasite strains. OHyW was detected by the mass transition from a parental ion (m/z 525.1945) to a product ion (m/z 393.1523). m^5^U was used as an internal control, using the mass transition from m/z 259.0930 to m/z 127.0508. One-way ANOVA corrected by Tukey’s multiple comparison tests was conducted (adjusted p-values: WT vs Tyw1a KO: 0.0021,Tyw1a KO vs Tyw1a addback: 0.01219, and WT vs Tyw1a addback: 0.223). The results of three biological replicates are shown. **C, D)** The frequency of metacyclogenesis, the percentage of the number of metacyclic cells relative to the total number of cells, induced in TAU 3AAG (**C**) and RPMI medium **(D)**. The parental cell line (Cas9), Tyw1a knockout (Tyw1a^-/-^), and Tyw1a add-back cell lines are shown. Experiments were performed with 5 biological replicates derived from different stocks of each cell line. The Cas9 strain expressing only Cas9 but not guide RNA was used as a control. Statistical analysis was performed using one-way ANOVA (RPMI) or two-way ANOVA (TAU 3AAG) with multiple comparisons test (for adjusted p values by Turkey multiple comparison correction: **** = 0.0001; *** = 0.001; ** = 0.01; * = 0.05; ns = not significant). **E)** Schematic illustration containing the abundance of Tyw1a and OHyW37 modification on tRNA^phe(GAA)^ in *T. cruzi*. The red arrow indicates downregulation of Tyw1a or OHyW37 modification in MT compared to EPI, while the blue arrow indicates upregulation in EPI. Reduced Tyw1a levels facilitate the metacyclogenesis process.

To test this hypothesis, we assess the impact of Tyw1a deletion on the differentiation from EPI to MT form. We generated EPI cell lines lacking Tyw1a using the CRISPR-Cas9 technique, along with the add-back cell lines (S4 Fig). We confirmed that the KO lineages do not express the Tyw1 enzyme, while the complemented parasite expresses Tyw1 (S4C Fig). Additionally, we confirmed that OHyW signal was eliminated in Tyw1 KO parasites and rescued in Tyw1 add-back strains by LC-MS analysis (Fig 4A and 4B). Subsequently, the parental strain (Cas9 strains), Tyw1 KO parasites (Tyw1^-/-^), and Tyw1 add-back parasites were induced to metacyclogenesis using two different differentiation strategies (TAU 3AAG and RPMI) [47,48]. In both conditions, the Tyw1 KO parasites exhibited more than a two-fold increase in the frequency of differentiation from EPI to MT compared to the parental strain (Cas9), while this increase in differentiation was partially or fully blocked by the ectopic expression of Tyw1a (Fig 4C and 4D). These results strongly suggest that Tyw1a and the synthesis of OHyW slow down *T. cruzi* differentiation. Given that the Tyw1 expression level and OHyW frequency were lower in the MT form, *T. cruzi* may facilitate differentiation from the non-infective EPI to the infective MT form through the suppression of Tyw1a expression and its consequent decrease in the OHyW levels (Fig 4E).

## Discussion

The profiles and biological roles of tRNA modifications remain largely unexplored across many organisms. In this study, we profiled tRNA modifications in different *T. cruzi* life forms and observed dynamic changes in the level of many tRNA modifications. We found that OHyW37, whose level was decreased in the non-replicative MT form, likely facilitates differentiation from the replicative EPI to the non-replicative MT form, suggesting that *T. cruzi* might control tRNA modification level to regulate the rate of differentiation. Other tRNA modifications that showed differential levels among different *T. cruzi* life forms in our data might contribute to the differentiation of life forms, or reflect responses to the distinct metabolic and environmental conditions encountered throughout the *T. cruzi* life cycle. tRNA-seq predicted the following tRNA modification sites: m^1^G9, acp^3^U20, m^3^C20, m^2^_2_G26, m^3^C32, mcm^5^s^2^U34, I34, OHyW37, m^1^G37, m^1^I37, ms^2^t^6^A37, f^5^C34, C-to-U34, m^3^CV13, acp^3^D47, m^1^A58, m^1^A59, C34, and C48. BLAST search found *T. cruzi* homologs of enzymes that create these modifications, supporting the mapping of these modifications. Some types of modifications generated by homologs of tRNA modifying enzymes in *T. cruzi* were not detected through tRNA-seq since tRNA-seq cannot detect modification that do not affect Watson-Crick base pairing during reverse transcription [32,49–52]. For example, m^5^C cannot be detected through tRNA-seq, although we found three homologs (Ncl1, Trm4Aa, and Trm4Ab) of the methyltransferase that generates m^5^C. To map these additional chemical modifications would be needed. Recently, in *T. brucei*, m^5^C was mapped through the bisulfide method [53], revealing that *T. brucei* has m^5^C at positions observed in other eukaryotes such as 34, 48, and 49. Since *T. cruzi* and *T. brucei* have the same set of homologs of m^5^C methyltransferases, *T. cruzi* may have the same profiles of m^5^C in tRNAs. Additionally, m^5^C was found at three more positions, C12, C50, and C60, in *T. brucei* tRNAs, further suggesting that the five C5- methyltransferases identified in *T. cruzi* (C4B63_23G207, C4B63_25G215, C4B63_81G44, C4B63_44g246 and C4B63_4g245) may be involved in catalyzing these modifications in its tRNAs. Further profiling with other complementary methods will be necessary for obtaining comprehensive tRNA modification maps of *T. cruzi*.

In *T. cruzi*, we detected misincorporation signatures at cytidine positions 34 and 48 (C34 and C48) by tRNA-seq in certain tRNA species, such as tRNA^Ser2 (CGA)^ and tRNA^Pro2 (CGG)^, respectively, that don’t correspond to known modifications in other organisms. C48 is frequently methylated to form m^5^C in eukaryotes [38]; however, this methylation does not produce RT-derived signatures due to its lack of direct effects on Watson-Crick base pairing [54,55]. While we cannot rule out the possibility that sequence context affects m^5^C to cause RT-derived signatures, C48 may undergo other modifications that have not yet been observed. tRNA-seq also detected misincorporation at C34 in multiple tRNA species. In *T. brucei*, C34 in other tRNA species undergoes modifications that cause RT-derived signatures, such as C-to-U editing [41]. It is possible that these modifications and editing occur in other tRNA species in *T. cruzi*. Further experiments, including mass spectrometry analysis of purified tRNAs, are necessary to identify these modification types.

One of the Trypanosoma-specific modifications is acp^3^D [29]. While many tRNA modifications showed a decrease in misincorporation only in the MT form, which is one of two non-proliferative forms, misincorporation signatures corresponding to acp^3^D47 showed a decrease in both non-proliferative forms, MT and TCT. acp^3^D47 is predicted to be present in 22 tRNA species, with over 23% of them showing reduced expression during the parasite’s non-proliferative stages. The 3-amino-3-carboxypropyl (acp) group is a highly conserved modification in both bacteria and eukaryotes, attached to the N3 atom of uracil (acp^3^U) at positions 20 and 47 [56–58]. In *T. cruzi*, we detected RT-derived signatures for acp3 at positions 20 and 47 on tRNAs; however, we predict that at position 47 this modification occurs on a dihydrouridine (D) rather than a uridine. The D base is not detected by tRNA-seq, as it does not induce misincorporation or premature termination during cDNA synthesis [32]. The prediction of modifications at the D47 base is based on two pieces of evidence: i. tRNAs with acp^3^ at position 47 within a D base instead of a U was detected in tRNA^Lys(TTT)^ from *T. brucei* [29]; ii. the presence of the homologous DUS3L, associated with the U47-to-D47 modification [59] along with the enzyme DTWD2 involved in acp3 [60] in *T. cruzi.* To our knowledge, no modifications were found at D, except for acp3 in only one tRNA species (Lys-TTT) from *T. brucei*. Thus, we hypothesize that the tRNA^Lys2(TTT)^ is also modified to acp^3^D47 in *T. cruzi*. The remaining 21 tRNAs, such as tRNA^Lys1A(CTT)^, tRNA^Asn1A(GTT)^ and tRNAVal1^(AAC)^, likely contain acp^3^U or acp^3^D at position 47.

*T. cruzi* has many paralogs of tRNA-modifying enzymes, which likely add complexity to the regulation of tRNA modification through several processes, including localizing modifications to distinct subcellular compartments, introducing modifications at distinct nucleoside positions, and differentially controlling modifications in the different parasite life forms. The presence of multiple modification enzyme paralogs in *T. cruzi* also highlights the intricate mechanisms regulating tRNA modifications and their potential impact on cellular processes. For example, the two paralogs of Abp140 (Abp140a and Abp140b) are likely responsible for 3-methylcytidine (m^3^C) in at least one of the following positions: C20, C32, or C47. In *T. brucei*, two paralogs, TRM140a (Tb927.10.1800) and TRM140b (Tb927.9.11750), have been identified as being associated with the m^3^C modification in tRNAs [61,62]. However, only TRM140a is capable of modifying the m^3^C32 position [62], strongly suggesting that its homolog in *T. cruzi*, Abp140b, has a preference for m^3^C32 modification, while Abp140a may target other sites. Abp140b, but not Abp140a, showed decreased levels in the MT relative to the EPI form. According to the tRNA-seq datasets, RT-derived signals in C20 and C32 change in opposite directions. In the MT form relative to EPI and TCT forms, the signals at C20 increased whereas the signals at C32 decreased. Such distinct dynamic changes of tRNA modification may be attributable to the differential regulation of paralogs of tRNA modifying enzymes.

Our results showed that Tyw1a/OHyW can control the differentiation frequency of *T. cruzi*. Suppressing the expression of Tyw1a enzyme and its associated modification OHyW promoted the transition from the non-infective EPI form to the infective MT form. One potential mechanism underlying this process is that the change in nutrient availability triggers a reduction of OHyW37 on tRNA^Phe(GAA)^, which could lead to the downregulation of certain proteins. During the metacyclogenesis, a reduction in global translation levels is crucial for EPI to MT differentiation [63]. Additionally, the MT form translates a narrower range of proteins compared to the EPI form [7] Therefore, the reduction of OHyW level potentially triggers a shift in the parasite’s gene expression required for the transition from the EPI to MT form.

One limitation of our study is the inability to distinguish between cytoplasmic and mitochondrial tRNAs. In *T. cruzi* both cytoplasmic and mitochondrial tRNAs are encoded in the nucleus and share identical sequences [39,40]. Therefore, sequence data represents tRNAs from both compartments. This may explain why the frequency of some RT-derived signatures is relatively low. For example, tRNA^Trp4(CCA)^ undergoes C-to-U editing at position 34 only in mitochondria but not in the cytoplasm. We observed low-level misincorporation of U at this position, likely reflecting the low ratio of mitochondrial tRNA to cytoplasmic tRNA. Additionally, differential changes between cytosolic and mitochondrial tRNAs may mask each other. In *T. brucei*, position 37 in tRNA^Phe(GAA)^ is modified to OHyW in the cytoplasm and wyosine in mitochondria [45]. Interestingly, the cytoplasmic Tyw1a is downregulated in the MT form, but mitochondrial Tyw1b is not changed, suggesting that this modification frequency is altered only in the cytoplasm. However, both OHyW and wyosine generate RT-derived signatures, and changes in one modification may be underestimated due to masking by the unchanged presence of the other. A modest change in the misincorporation signal at this position between EPI and MT forms could be attributed to this issue. Separating mitochondrial and cytoplasmic fractions during sample preparation will help resolve this issue. Another potential confounding factor is the use of the distinct culture conditions for induction of differentiation. Some of the observed changes in tRNA modifications may be attributed to the change of environments independent of differentiation processes.

Overall, our results suggest that tRNA modifications are differentially regulated across the life stages of *T. cruzi* and identified specific tRNA-modifying enzymes linked to the parasite’s survival and differentiation. The data generated in this work lays the foundation for a deeper understanding of novel mechanisms of regulation of protein synthesis in trypanosomes.

## Materials and methods

### Cell culture

The EPI forms of *T. cruzi* (Dm28c or CL Brener strains) were cultured at 28°C in Liver Infusion Tryptose (LIT) medium supplemented with 10% fetal bovine serum (FBS - Vitrocell), 0.4% glucose, 0.1 µM hemin, and 60 mg/mL penicillin G, following Camargo et al., 1964 [64]. The MT forms were obtained using the method described following Contreras et al., 1985 [47], with some modifications. Briefly, EPIs in the exponential growth phase (4x10⁶ parasites/mL) were cultured for four days until reaching the stationary phase (5-6x10⁷ parasites/mL). The parasites were then resuspended at a concentration of 5x10⁸ parasites/mL in Triatomine Artificial Urine (TAU) medium (190 mM NaCl, 17 mM KCl, 2 mM CaCl₂, 2 mM MgCl₂, and 8 mM phosphate buffer, pH 6.0) and incubated for 2 hours at 28°C. Afterwards, the parasites were diluted to 5x10⁸/mL in TAU 3AAG medium (containing 10 mM L-proline, 50 mM L-glutamate, 2 mM L-aspartate, and 10 mM glucose) and kept at 28°C in a CO₂ incubator. The MTs were collected from the culture supernatant after 76 hours of incubation and purified using DEAE-Cellulose resin (Sc-211213). The TCT forms of the Dm28c strains were collected from the supernatant of LLCMK2 cells one-week post-infection (at a ratio of 1:40, cells to parasites), following the method described by Nogueira et al, 1976 [65]. The LLCMK2 cells were cultured in DMEM (Gibco) supplemented with 3.7 g/L NaHCO₃, 0.059 g/L penicillin G, 0.133 g/L streptomycin, and 10% fetal bovine serum (FBS) at 37°C with 5% of CO₂. The HEK293 cells were cultured in DMEM (Gibco) supplemented with 0.059 g/L penicillin G, 0.133 g/L streptomycin, and 10% fetal bovine serum (FBS) at 37°C with 5% of CO₂. The HCT116 cells were cultured in McCoy’s 5A medium (Gibco) supplemented with 0.059 g/L penicillin G, 0.133 g/L streptomycin, and 10% fetal bovine serum (FBS) at 37°C with 5% of CO₂.

### tRNA sequencing, processing and analysis

tRNA-seq for TCT samples was conducted together with EPI and MT samples as previously described [12]. Briefly, total RNA from *T. cruzi* life forms from the Dm28c strain (in biological duplicates) was isolated using Trizol (Invitrogen), followed by the tRNAs purification and sequencing based on the method of Kimura et al., 2020 [32], with slight modifications described at Silva et al., 2024 [12]. The cDNAs from tRNA of TCT were synthesized with 5 pmol of TGIRT-III (InGex #TGIRT10) in 100 mM Tris-HCl (pH 7.5), 0.5 mM EDTA, 450 mM NaCl, 5 mM MgCl₂, 5 mM DTT, 1 mM dNTPs, and 1.25 pmol of the primer, followed by incubation at 60°C for 1 hour. The primer sequence used for the reverse transcription reaction was as follows:/5Phos/AGATCGGAAGAGCGTCGTGTAGGGAAAGAG TGT/iSp18/CAAGCAGAAGACGGCATACGAGATCG. Subsequently, cDNAs were circularized with 50 U of CircLigase II ssDNA ligase (Epicenter/Lucigen #CL9021K) at 60°C for 1 h, with an additional enzyme addition. Circularized cDNAs were amplified using Phusion High-Fidelity DNA Polymerase (NEB #M0530S) using the following condition: 98°C for 30 sec; (8 × : 98°C for 10 sec, 60°C for 10 sec, and 72°C for five sec). The reverse primer used was CAAGCAGAAGACGGCATACG in all reactions, while index-specific forward primers were as described in [12,32]. The tRNA samples were sequenced on an Illumina NextSeq 1000 system (single-end). FASTQ files from EPI and MT were obtained from PRJNA112437 and re-reprocessed together with FASTQ files from TCT. The adapter sequences (AGATCGGAAG) were removed from the FASTQ files using the cutadapt v.2.8 tool [66], followed by quality trimming using TrimmomaticSE v.0.39 [67] with HEACROP:2 settings. The reads were then mapped using Bowtie 1.3.1 [68], and mpileup files were generated using the samtools mpileup command (options: -A –ff 4 -x -B -q 0 -d 10000000 -f). The frequency of misincorporation was calculated in each mpileup file using a Python script to find the tRNAs Modifications [32]. The coverage values of the 5’ termini of mapped reads were obtained using the bedtools genomecov command (option: -d −5 -ibam). To calculate the termination frequency, the number of 5’ termini at any position was divided by the total number of mapped 5’ termini at the given position, along with all upstream positions (5’ side) [32]. The frequencies of misincorporation and termination were visualized using R (v.4.3.1) in a heatmap.

In this study, we use misincorporation signals to predict modification sites. According to tRNA modifications in *Homo sapiens* and *Saccharomyces cerevisiae*, eukaryotes have several modifications at specific positions that strongly affect Watson-crick base pairing, thereby induce misincorporation: C: m^3^C at position 20 and 32, U: acp^3^U at position 20 and acp^3^D at position 47(D), G: m^1^G at position 9 and 37, m^2^_2_G at position 26, A: I at position 34, m^1^I at position 37, and m^1^A at position 58 and 59. When we observed misincorporation signals at these bases at these positions, at a frequency higher than an arbitrary threshold (5%) in both two replicates of EPI states, we assigned the modifications. We also used specific information from other references to assign a couple of modifications, which include OHyW at position 37 in tRNA-Phe (PMID 20739293) and ms^2^t^6^A at position 37 in tRNA-Lys2 (PMID 22040320 and 27913733), and mcm^5^s^2^U at position 34 in several tRNA species (PMID 22040320). It is noteworthy that we did not rule out that a subset of these modified sites contains different types of modifications.

### Mass spectrometry

180 ng of tRNA fractions purified from the total RNA extracted from *E. coli* grown in LB medium, *T. cruzi* in the exponential phase, and HEK293 and HCT116 cells were digested by 0.5 unit of Nuclease P1 (US Biological) and 0.1 unit of Phosphodiesterase I (Sigma) in 20 µl aliquot containing 50 mM NH_4_OAc pH 5.3, and 1 mM ZnCl_2_, at 37°C for 1 hr, followed by the addition of 2 µl of 1 M Tris-HCl pH 8.0 and 1 µl of 1 U/µl phosphatase (Sigma) for dephosphorylation at 37 °C for 30 min. After removal of the enzymes by using a 10K filter column, 16 µl of nuclease digest was loaded to LC-MS. UltiMate3000RSLCnano ultra-high-performance liquid chromatography (uHPLC) system (Thermo Scientific) bearing Acquity UPLC BEH C18 column (150 × 2.1 mm, 1.7 µm, 130 Å, Waters) at 40 °C with a flow rate 0.2 ml/min with a solvent system consisting of 5 mM NH_4_OAc pH 5.3 (Buffer A) and 100% Acetonitrile (Buffer B). The gradient of acetonitrile was as follows: 0–2 min: 0.5% B, 2–13 min: 0.5-60.5% B, 13–17 min: 60.5-98.0% B, 17–20 min: 98% B, 20–21 min: 98-0.5% B, 21–25 min: 0.5% B. The eluent was ionized by an electrospray ionization source and injected into a ZenoTOF 7600 (SCIEX) with the positive mode of MRMhr scan. The voltages and source gas parameters were as follows: spray voltage; 5500 V, source name; TurbolonSpray, curtain gas; 35, CAD gas; 7, ion source gas 1; 40 psi, ion source gas 2; 50 psi, ion source temperature; 450 °C, and Collision energy; 7 V. The list of ions for tSIM analyses are given in S3 Table. The data was analyzed and visualized using Skyline version 25.1.0.142. Nucleoside peaks are identified using Human and *E. coli* samples as references. Three methyl guanosines (m^7^G, m^2^G, and m^1^G), three methyl adenosines (m^1^A, m^6^A, and m^2^A), and two methyl cytidines (m^3^C and m^5^C) were detected at the same mass transition but eluted as separated peaks. Based on the order of elution in the references using C18 column, we assigned the modifications to the peaks.

### Identifying tRNA-modifying enzymes in Trypanosoma

A FASTA file containing the amino acid sequences of 216 enzymes involved in tRNA modifications in prokaryotes and eukaryotes was obtained from MODOMICS [56] except for cysteine desulfurase from *T. brucei* (Tb927.11.1670) [69] and a DTW domain containing protein (DTWD2 -Tb927.3.4690) [70]. This file includes 217 of known and putative tRNA modifying enzymes present in different organisms. Subsequently, the Local Alignment Search Tool (BLAST) was applied to the genome of the Dm28c strain version 2018 (Available at https://tritrypdb.org/tritrypdb/app/workspace/blast/new) to identify the homologous genes in *T. cruzi*. Alignments with an E-value ≤ 1E-10 were considered to be homologous genes in *T. cruzi.* The tModBase database [38] was used as an auxiliary tool to identify the modification sites on tRNA for the enzymes characterized in this study. Orthologous tRNA-modifying enzymes in *T. brucei* TREU927 were identified using the ‘strategies’ and “orthology phylogenetic profiles” tools available in TriTrypDB (https://tritrypdb.org/tritrypdb/app). The cellular localization of enzymes associated with tRNA modifications and their impact on survival in *T. brucei* was examined using the platform (http://tryptag.org/) [36]and by consulting the list of proteins provided by Alsford et al., 2011 [35], respectively.

### Translation rates in EPI and MT forms

Ribo-seq in EPI and MT forms of *T. cruzi* were obtained from the Sequence Read Archive (SRA) repository, project ID: PRJNA260933, provided by Smircich et al., 2015 [7]. Transcript per million (TPM) values for the tRNA-modifying enzymes in *T. cruzi* life forms identified in this study were obtained from the table of global translatome values for the EPI and MT forms provided by Silva., et al 2025 [12].

### Generation of null mutants using CRISPR-Cas9 and addback cell lines

Parasites CL Brener (EPI) expressing the Cas9 protein endogenously were used for genome edition [71]. Primers listed in S4 Table were designed using the EuPaDGT tool [72] to obtain the sgRNA sequences for targets: Tyw1a: TcCLB.508277.60 and TcCLB.503543.4. The sgRNAs were amplified by PCR using Platinum Taq DNA Polymerase (Invitrogen). For amplifying the DNA donor, 30 bp homologous sequences located up and downstream of sgRNA binding sites of the genes of interest were added in the template primers to replace the genes of interest with the blasticidin or puromycin resistance genes. Primers were designed manually (in S4 Table) and the plasmids pJET-blast or pT-puro [71] were used as templates for the PCR using 3 U Platinum Taq DNA Polymerase High Fidelity (Invitrogen) in a total reaction volume of 120 μL. To generate addback cell lines, the coding sequences of Tyw1a were amplified by PCR using specific primers flanked by restriction sites (S4 Table). The Tyw1a PCR product was gel purified using QIAquick Gel Extraction Kit according to manufacturer’s instructions, digested with EcoRI Fast digest and XhoI Fast digest (Thermo Fisher Scientific) and ligated with similarly digested pTEX_puroR treated with FastAP Thermosensitive Alkaline Phosphatase (Thermo Fisher Scientific). Before transfection, sgRNA and DNA donor PCR products and 25 μg of the plasmids were purified, precipitated and resuspended in 5 μL of sterile ultrapure water each. To parasite transfections, 4 × 10^7^ of EPI were treated with 20 mM hydroxyurea (Sigma) for 18 hours [73]. Then, the parasites (4 × 10^7^) were resuspended in 1 mL of transfection buffer (90 mM sodium phosphate, 5 mM potassium chloride, 0.15 mM calcium chloride, 50 mM HEPES, pH 7.2), along with the DNA to be transfected in a 0.2 cm cuvette (BioRad) using the Nucleofector 2b device (Lonza) with a single pulse using program U-033. The modified parasites were selected in the presence of antibiotics and subsequently cloned in 96-well plates. Null and addback parasites lineages were confirmed by PCR using specific pairs of primers described in S4 Table. Agarose gel electrophoresis was used to visualize the positive PCR amplifications.

### Western blot

Total protein extracts were separated by 10% polyacrylamide gel electrophoresis (SDS-PAGE) and transferred to a nitrocellulose membrane (BioRad) using the TransBlot Turbo system (BioRad), as described by [74] and following the manufacturer’s instructions. The membrane was blocked for 1 hour in PBS-T (0.1% PBS-Tween-20) solution containing 5% skim milk. After blocking, the membrane was incubated with a primary anti-6xHisTag monoclonal antibody (Invitrogen, MA121315) at a 1:1000 dilution in PBS-T solution containing 3% skim milk, with gentle agitation overnight at 4° C. Next, the membrane was washed three times for 5 minutes with PBS-T. Subsequently, the membrane was incubated for 1 hour with the secondary antibody conjugated to horseradish peroxidase (HRP, dilution 1:2000; GE Healthcare), followed by additional washes as described above. Finally, the assays were developed using chemiluminescence with the ECL Super Signal West Pico Chemiluminescent Substrate (Thermo Scientific) according to the manufacturer’s instructions and visualized using the ChemiDoc Imaging System transilluminator (BioRad).

### RT-qPCR

Total RNA extractions from *T. cruzi* null and addback lineages were performed using Trizol (Invitrogen) according to the manufacturer’s instructions. The reverse transcription reaction was performed using RNA previously treated with DNAse I, Amplification Grade (Invitrogen) and the SuperScript III First-Strand Synthesis (Invitrogen) using 50 ng Oligo(dT)12–18 (Invitrogen), following the manufacturer’s instructions. For each sample, a control without reverse transcriptase (-RT) was prepared. qPCR reactions were performed using the 5x HOT FIREPol Probe Universal qPCR Mix (SolisBioDyne) including 4 µL of reagent mix, 400 nM of primers, 1x SYBR Green, 1 µL of cDNA, and nuclease-free water. All samples were analyzed in technical triplicate, with parallel reactions performed without cDNA or with -RT samples as negative controls. Amplification conditions were carried out on the StepOnePlus Real-Time PCR system (Applied Biosystems) as follows: initial denaturation at 95°C for 10 minutes, followed by 40 cycles of denaturation at 94 °C for 15 seconds and annealing at 60 °C for 1 minute, and a final melting curve from 60 °C to 95 °C. Primers were designed using Primer3 (https://primer3.ut.ee/) [75] the sequences of Tyw1a, and GAPDH (TcCLB.506943.50) as templates (S4 Table). The gene glyceraldehyde 3-phosphate dehydrogenase (TcGAPDH) was used as a normalizer for calculating relative expression. Data were analyzed using the StepOne Software v2.3. Variations in transcript expression were determined using the 2(-ΔΔCt) method [76].

### *In vitro* metacyclogenesis for *T. cruzi* null and addback strains

To conduct *in vitro* metacyclogenesis assays, two different methods were used. In the first method [47], EPIs in the exponential growth phase were used to initiate a culture with an initial concentration of 5 x 10^6^ parasites ml^-1^, which was maintained in LIT as previously described for 6 days. After, the parasites were washed with PBS 1x and then transferred to TAU medium (190 mM NaCl, 17 mM KCl, 2 mM MgCl_2_, 2 mM CaCl_2_, 8 mM potassium phosphate buffer, pH 6) at a concentration of 5 x 10^7^ parasites ml^-1^ and maintained for two hours at 28°C. The parasites were subsequently transferred to TAU 3AAG (TAU supplemented with 10 mM glucose, 2 mM aspartic acid, 50 mM glutamic acid, 10 mM proline) and maintained for 8 days in a CO_2_ incubator at 28°C. The presence of MT was checked daily by counting in a Neubauer chamber. In the second protocol [48], 1 mL of EPI culture in the exponential growth phase (5 x 10^7^ cell ml^-1^) was transferred to a culture flask containing 10 mL of RPMI (Vitrocell; prepared according to the manufacturer’s instructions, without the addition of FCS and pH adjustment). The flasks were kept undisturbed at a 30-degree angle in a CO_2_ incubator at 28°C for 8 days. After 8 days, the number of MT in the supernatant was counted using a Neubauer chamber.

### Graphs and statistical analysis

All graphs and statistical analysis were performed using GraphPad Prism v10.

## Supporting information

S1 Table Identification of tRNA-modifying enzymes in *T. cruzi* genome. **A)** List of the enzymes (gene ID) associated with tRNA Modifications in several eukaryotes and prokaryotes. **B)** BLAST results containing the best hit of the tRNA-modifying enzyme found in the *T. cruzi* genome, along with the associated tRNA Modifications. The proteins showing E-value ≤ 1e-10 were considered as tRNA modifying enzyme homologs. **C)** List of tRNA-modifying enzymes in *T. cruzi* and their orthologs in *T. brucei*. **D)** List containing the tRNA-modifying enzymes analyzed in public RIT-seq data [35] linked with *T. brucei* fitness during the differentiation of procyclic to bloodstream trypomastigote forms. **E)** Fold change and adjusted p-values for tRNA-modifying enzymes between EPI and MT forms obtained with Ribo-seq data.
(XLSX)

S2 Table Characterization of tRNA modifications in *T. cruzi.* **A)** Gene ID and genomic localization of all tRNA species present in the *T. cruzi* genome. **B)** List containing all tRNA Modifications either identified or predicted using tRNA-seq and BLAST, respectively. **C**) Values of proportion of misincorporation from tRNA-seq.
(XLSX)

S3 Table Parameters and source data for mass spec analyses. **A**) The list of the ions detected by LC-MS with the mass-to-charge ratios of parental ions and product ions. B) Source data related to Fig 2A.
(XLSX)

S4 Table Primer sequences and PCR conditions were used for the construction and validation of genetically modified *T. cruzi* parasites generated in this study. (XLSX)

S5 Table Abbreviations list. (XLSX)
**S1 Fig.** **Mass chromatograms detecting tRNA modifications in two replicates of *T. cruzi*, HEK293 cells, HCT116 cells, and *E. coli*.** Mass-to-charge ratios are listed in S3 Table.
(PDF)
**S2 Fig.** **Quality check of tRNA-seq data in *T. cruzi* life forms (EPI, MT and TCT).** **A)** Number of total reads and reads mapped to the *T. cruzi* genome are shown. Reads were exclusively mapped to tRNA sequences. **B)** Illustrative example of high sequence quality in the EPI R1 sequencing data after adaptor trimming using Phred score as a parameter. R1= replicate 1 and R2= replicate 2.
(PDF)
**S3 Fig.** **tRNA-seq results from two biological replicates derived from different strain stocks.** **A)** Termination signatures of EPI replicate 1. **B)** RT-derived signatures (Misincorporation and Termination frequencies) in the EPI R2 (second biological replicate) sample. **C)** Correlations of misincorporation frequencies in identified modified sites listed in Table S2C between two replicates of three differentiation forms. R-square values of Pearson correlation tests are shown.
(PDF)
**S4 Fig.** **Generation and confirmation of Tyw1a knockout and add-back cell lines.** **A)** Schematic shows the strategy used to replace the coding sequence of Tyw1a with blasticidin and puromycin resistance genes using the CRISPR-Cas9 technology as well as the oligonucleotides used for genotyping. **B)** Genotyping of five individual clones of the Tyw1a^-/-^ EPI cell line using the PCR strategies described in A. **C)** Graph shows relative Tyw1a mRNA levels of five individual clones of the TcTyw1a^-/-^ EPI cell line compared with the Cas9 parental cell line, assessed by RT-qPCR. **D)** Graphic representation of the vector used for generation of the Tyw1a addback cell lines. This vector allows the episomal expression of Tyw1a with a 6xHis tag in the C-terminal. **E)** Confirmation Tyw1a cloning in the 6xHis_pTEX_hygro vector using *BamH*I and *Hind*III restriction sites. **F)** Western blotting shows the expression of Tyw1a-6xHis (~94 kDa) in three individual clones of the addback EPI cell lines. The loading control was the total proteins on the gel stained with trichloroethanol (TCE).
(PDF)
